# Effects of salinity on flowering, morphology, biomass accumulation and leaf metabolites in an edible halophyte

**DOI:** 10.1093/aobpla/plu053

**Published:** 2014-09-01

**Authors:** Yvonne Ventura, Malika Myrzabayeva, Zerekbay Alikulov, Rustem Omarov, Inna Khozin-Goldberg, Moshe Sagi

**Affiliations:** 1 The Albert Katz Department of Dryland Biotechnologies, The Jacob Blaustein Institutes for Desert Research, Ben-Gurion University, PO Box 653, Beer Sheva 84105, Israel; 2 Department of Biology and Biotechnology, The L.N. Gumilyov Eurasian National University, 5 Munaitpasov Street, 473021 Astana, Kazakhstan

**Keywords:** Antioxidant compounds, *Crithmum maritimum*, flowering, germination, omega-3 polyunsaturated fatty acids, salinity.

## Abstract

The fresh water shortage in agriculture is an increasing problem worldwide, therefore the possibility of cultivating crops under saline conditions is of high importance. *Crithmum maritimum*, a halophytic plant naturally found on the rocky coastlines of the Atlantic Ocean and the Mediterranean Sea, has a long history of human consumption and was recently suggested as a cash crop for biosaline agriculture. In the present study, we compared the responses of different genotypes originating from France, Portugal and Israel to moderate saline irrigation (up to 100 mM NaCl). The genotypes varied greatly in the onset of flowering, their leaf appearance, growth habits and leaf metabolite content. Both Atlantic genotypes (from France and Portugal) flowered earlier than those from the Mediterranean, but the number of inflorescences decreased with salinity. Irrigation with 50 and 100 mM NaCl led to a reduction in biomass production in both the Israeli and the Portuguese genotypes, while the French genotype was found to produce maximum leaf yield at 50 mM NaCl. With increasing salinity, salt was accumulated by the plants, as indicated by increasing electrical conductivities of the leaf extracts. Concomitantly, antioxidant compounds (such as ascorbic acid), total polyphenols and ureides responded to salinity in a genotype-dependent manner; either they increased, decreased or were unaffected. Notably, the total fatty acid concentration increased with salinity in both Mediterranean genotypes, reaching 2.7 and 2.4 % total fatty acids (on a dry weight basis) at 100 mM NaCl. Moreover, the proportion assigned to omega-3 fatty acids in these genotypes was higher than in their Atlantic counterparts at the highest salinity tested. Our results highlight the variations existing among *C. maritimum* genotypes from different origins regarding salt-induced changes in plant growth, flowering behaviour and leaf metabolites with nutritional value. Thus, genotypic characteristics should be taken into account when evaluating a wild plant species for future crop cultivation.

## Abstract (precis)

Cultivating crops under saline conditions is of high importance due to global fresh water shortage for irrigation. *Crithmum maritimum* is a halophytic plant that has a long history of human consumption and was suggested as a cash crop for biosaline agriculture. Our results highlight variations existing among *Crithmum maritimum* genotypes from different geographic origins regarding salt-induced changes in plant growth, flowering behavior and leaf metabolites with nutritional value. Our results indicate that genotypic characteristics should be taken into account when evaluating wild plant species for future crop cultivation.

## Introduction

Salinization is one of the major environmental constraints limiting crop production (Maggio *et al.* 2011). This phenomenon is particularly expressed in arid and semiarid regions due to high evaporation and low precipitation rates. Paradoxically, irrigation in these extreme environments has led to the accumulation of salts in the uppermost soil layers of arable lands. Thus, to date, large areas of freshwater-irrigated lands have suffered from salt and water build-up in the root zone (Yensen 2006; Rozema and Flowers 2008). Halophytes are equipped with well-defined adaptive mechanisms that enable them not only to withstand periodical high salinity, but also to complete their entire lifecycles at high salinities (Flowers *et al.* 2010). The tolerance of halophytes to salinity relies mainly on the controlled uptake of ions and the vacuolar compartmentalization of Na^+^, K^+^ and Cl^−^ with the achievement of an osmotic balance between vacuoles and cytoplasm by synthesis of osmotically active metabolites (Sagi *et al.* 1997; Flowers and Colmer 2008). Conversely, the increased synthesis of these organic compounds and the active transport of toxic ions across the vacuolar membrane have a considerable energetic cost, resulting in growth retardation and ultimately in the reduction of productivity (Greenway and Munns 1980; Zhu 2001). Nevertheless, halophytes are able to generate economic yields, although exposed to salt stress conditions (Koyro *et al.* 2011; Ventura *et al.* 2011*a*).

When plants are subjected to environmental stresses, including salinity, the production of reactive oxygen species may disrupt the integrity of cellular membranes and the activities of various enzymes resulting, eventually, in plant damage (Zhu 2001). To counteract the salt-mediated oxidative stress, plants up-regulate antioxidative enzymes and production of small non-enzymatic molecules [e.g. ascorbate (ASC), flavenoids and polyphenolic compounds]. High levels of antioxidants were found to be associated with oxidative stress tolerance in glycophytes and halophytes (Türkan and Demiral 2009). In addition, ureides, which are nitrogen-rich compounds with antioxidant activity, may play an important role during nitrogen transport under stressful conditions (Brychkova *et al.* 2008; Ventura *et al.* 2010). Enhanced tolerance to salt stress can be further achieved by changes in the saturation of fatty acids in the chloroplast membrane (Allakhverdiev *et al.* 1999), which mainly comprises α-linolenic acid (18 : 3ω3) and linoleic acid (18 : 2) in many plants (Simopoulos 2004).

*Crithmum maritimum* (Apiaceae) is a wild halophyte growing on the rocky coast lines of Western Europe and the Mediterranean. This plant species is well known for its high content of antioxidant compounds, and its leaves have been consumed, fresh or pickled, by humans for many years (Meot-Duros and Magné 2009; Atia *et al.* 2011). *Crithmum maritimum* was listed as a salt-tolerant species with the potential to be developed into a cash crop halophyte (Yensen 2006; Atia *et al.* 2011). To date, halophytes have had a broad field of utilization, ranging from consumption, through ornamental applications and renewable energy sources, to environmental protection (Koyro *et al.* 2011). An initial step in the development of a halophytic species as a cash crop includes the primary screening for its agronomic potential (Koyro *et al.* 2006).

Most publications investigating *C. maritimum* have used plant material collected from the wild, referring to specific sites, environments and seasons of sampling and therefore comparing only a narrow range of local genotypes (Cunsolo *et al.* 1993; Guil-Guerrero and Rodríguez-García 1999; Ben Amor *et al.* 2005; Özcan *et al.* 2006; Meot-Duros and Magné 2009). In the current study, we investigated the potential of four genotypes of *C. maritimum* for dual application: as an ornamental crop and as a leafy vegetable crop, both for saline agriculture. We determined leaf and flower appearance, biomass production and changes in nutritional metabolites in response to increasing salinity.

## Methods

### Sample collection and experimental setup

Seeds of two *Crithmum* genotypes were collected at two locations along the Mediterranean Sea shore in Israel and designated HB and IS. Two additional genotypes, originating from the Atlantic coasts of southern Portugal (PO) and Brittany in France (FR), were compared with their Mediterranean counterparts. The collected seeds were stored in a cold room at 4 °C for ∼2 months until sowing. All experiments were carried out in a temperature-controlled greenhouse located at Ben-Gurion University in Beer Sheva, Israel. Summer temperatures were kept <33 °C, using a cooling system, while in the winter the greenhouse was heated when temperatures dropped <20 °C. Midday photosynthetic photon flux density in the greenhouse was 300–500 μmol m^−2^ s^−1^. Seeds were germinated in 9-cm diameter Petri dishes on a double layer of filter paper (Whatman No. 1, Maidstone, Kent, UK) at room temperature (25 °C ± 3) using distilled water. Germinated seedlings of similar size were transferred to 12-cm diameter plastic pots filled with commercial potting mixture HR-1, composed of peat, tuff and synthetic sponge, including slow release fertilizer (Shacham Givat Ada, Ltd, Israel) and grown in the same greenhouse. Two weeks after plant transfer salt treatments (0, 50 and 100 mM NaCl) were applied by gradually (over 2 weeks) increasing the salt concentrations until the final concentration was reached. Plants were irrigated three times a week with the respective salt solution supplemented with commercial N–P–K fertilizer (20–20–20+microelements; Haifa Chemicals Ltd, Haifa, Israel) at a final concentration of 40 ppm nitrogen. Each salt concentration comprised five plants per genotype.

### Growth parameters

Plants were grown for ∼2.5 months and then harvested by cutting the green biomass ∼2 cm above the pot. Immediately after harvest, total shoot biomass per harvested pot unit was recorded. The dry weight was obtained after drying the plant material at 70 °C for 48 h and per cent dry weight was calculated. Leaves were photographed to document their morphology. Complete plant re-growth occurred approximately within a 1-month time period after the harvest (data not shown).

### Flower appearance

Appearance of inflorescences was recorded for the first time ∼2.5 months after the leaf harvest (months after harvest, MAH) and their number was recorded. The number of flowering branches was confirmed at two additional time points, 3.5 and 4.5 MAH. The data represent one of two similar experiments conducted during the same season.

### Evaluation of leaf constituents

Fresh leaf samples were collected before flower initiation, immediately snap-frozen in liquid nitrogen and stored at −80 °C until further evaluation. Extraction was performed as described previously by Ventura *et al.* (2011*a*). Briefly, the frozen samples were homogenized on ice at a 1 : 4 (w/v) ratio with doubly distilled water, centrifuged at 17 500 *g* for 20 min at 4 °C and filtered through Whatman filter paper No.1. Electrical conductivity (EC) was measured in the filtered water extract with a conductivity meter (CyberSan 500 Con, Eutech Instruments, Singapore), and total soluble solids (TSS [%]) were determined using a digital refractometer (Atago PR-1, Atago Co. Ltd, Japan).

### Determination of reduced ascorbate and dehydroascorbate

Ascorbate determination followed the procedure described by Law *et al.* (1983). Frozen plant tissue was homogenized with 5 % *meta*-phosphoric acid at a ratio of 1 : 5 (w/v). The extract was centrifuged at 14 000 *g* for 20 min at 4 °C (Z 233 MK-2, HERMLE) and the supernatant was collected. Reduced ASC was assayed in 5 mM ethylenediaminetetraacetic acid in 150 mM sodium-phosphate buffer (pH 7.4). Dehydroascorbate (DHA) was reduced to ASC prior to determination by the addition of 10 mM dithiothreitol (DTT), the excess of which was oxidized after 15-min incubation by the addition of 0.5 % *N*-ethylmalemide. The colorimetric assay was carried out at 37 °C for 1 h after the addition of a mixture containing 10 % trichloroacetic acid, 44 % *o*-phosphoric acid, 4 % α,α-dipyridil in ethanol and 3 % aqueous ferric chloride solution at a ratio of 2 : 2 : 1 : 1 (v/v). Light absorbance of the resultant samples was measured at 525 nm. Dehydroascorbate was calculated by the difference between total ASC and reduced ASC.

### Total polyphenol determination

Total polyphenols were determined according to Singleton and Rossi (1965). Frozen leaf samples were extracted with 100 mM sodium-phosphate buffer (pH 7) at a 1 : 4 (w/v) ratio using a mortar and pestle. Resulting extracts were centrifuged for 20 min, 14 000 *g* at 4 °C (Z 233 MK-2, HERMLE). Aliquots of the collected supernatant were assayed with 2 N Folin–Cioclateur reagent (Sigma) and 35 % Na_2_CO_3_, by 60-min incubation at 30 °C in a water bath. The absorbance of the resulting colour was measured at 730 nm (JASCO, model V-530) against a known standard of gallic acid. Total polyphenols were expressed in mg 100 g^−1^ FW as gallic acid equivalents (GAE).

### Determination of the ureides, allantoin and allantoate

Frozen leaf material was ground with 80 % ethanol at a ratio of 1 : 4 (w/v). The plant extract was centrifuged as described above. The collected supernatant was used to determine ureides according to Vogel and van der Drift (1970). For allantoin (ALN), the sample was boiled at 100 °C in 0.5 M NaOH for 8 min, then neutralized with 0.65 N HCl and boiled again for 4 min. Subsequently, 0.4 M P-buffer (Na_2_HPO_4_–KH_2_PO_4_) pH 7.0 and 18 mM phenyl hydrazine were added. Finally, the assay was mixed with concentrated ice-cold HCl and 50.6 mM potassium ferricyanide. Absorbance was determined at 535 nm. For allantoate (ALT) determination, 0.15 N HCl was added to the ethanolic extract and the assay mixture was boiled for 4 min at 100 °C. After cooling down, the assay was continued from the step of P-buffer addition as described for ALN.

### Fatty acid profile

To determine the fatty acid profile and content, young shoot tips and leaves were dipped for 30 s in chloroform to remove cuticular waxes prior to lyophilization. The lyophilized plant material was then ground into powder and transmethylated with 2 % sulfuric acid in dry methanol at 80 °C over 1.5 h in an argon atmosphere. Heptadecanoic acid (C_17_) (Sigma Chemical Co., St Louis, MO) was added as an internal standard. Fatty acid methyl esters (FAMEs) were then extracted with *n*-hexane, and their profiles were identified as previously described on a Thermo Ultra gas chromatograph equipped with autosampler, PTV injector, FID detector and a Zebron GC column (ZB-WAXplus 30 mL × 0.32 mm ID × 0.25 µm df), using authentic standards (Sigma Chemical Co.) (Ventura *et al.* 2011*a*).

### Statistical analysis

Statistical analysis was performed by two-factor analysis of variance (ANOVA) for all parameters by comparing salinity and genotype as main factors using the JMP In 5.0.1a software package (SAS Institute, Inc., Campus Drive, Cary, NC, USA). Subsequently, mean comparisons were performed according to Student's *t*-test with the same software.

## Results

### Growth parameters and plant appearance

**Figure 1.**
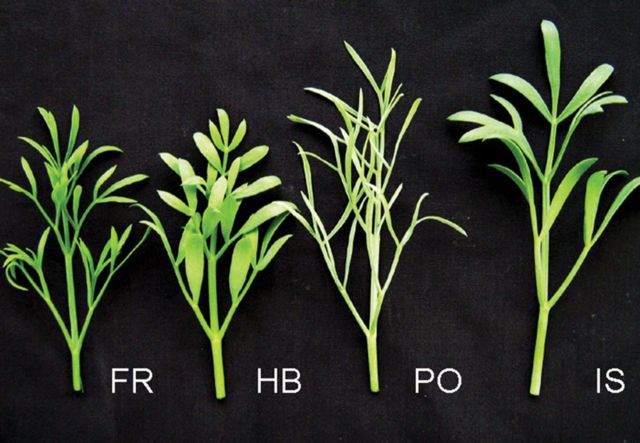
Leaf appearance of four *C. maritimum* genotypes: FR, HB, PO and IS.

Four genotypes of *C. maritimum* were investigated for their potential as cash crop halophytes with a dual function, as ornamental flowering plants for landscaping and as leafy vegetables with nutritional value. All genotypes could be easily distinguished by their leaf appearance; PO had a very narrow leaf lamina, while HB could be identified by its broad lamina of single leaflets (Fig. 1). IS and FR were intermediate, with FR being closer to its narrow-leaved Atlantic counterpart.

**Figure 2.**
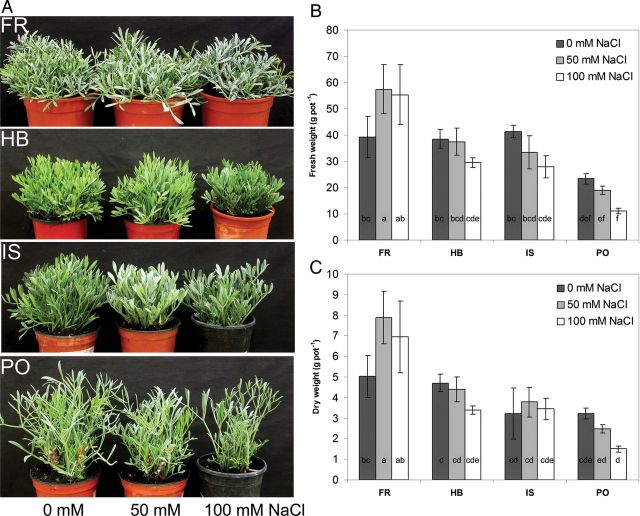
Effect of salinity on plant morphology (A), fresh weight (B) and dry weight (C) in four *C. maritimum* genotypes. Values denoted by different letters are significantly different, *P* < 0.05.

The highest biomass was produced by FR, followed by HB, IS and PO, which produced significantly less fresh weight than all other genotypes. Salinity significantly increased fresh and dry biomass only in the FR genotype at 50 mM NaCl, indicating its salt tolerance (Fig. 2B and C). Genotypes FR, HB and IS exhibited a compact growth habit, while PO presented a loose appearance (Fig. 2C), probably due to its long petioles and narrow leaf lamina.

### Flowering pattern

In order to test the multi-potential of *C. maritimum* as a leafy vegetable and ornamental crop, leaf metabolites were determined in leaves sampled before the onset of flowering. The fast re-growth, during ∼1 month, to the plant size before the harvest (data not shown) enabled the investigation of the continuous plant development and subsequent flower appearance.

**Figure 3.**
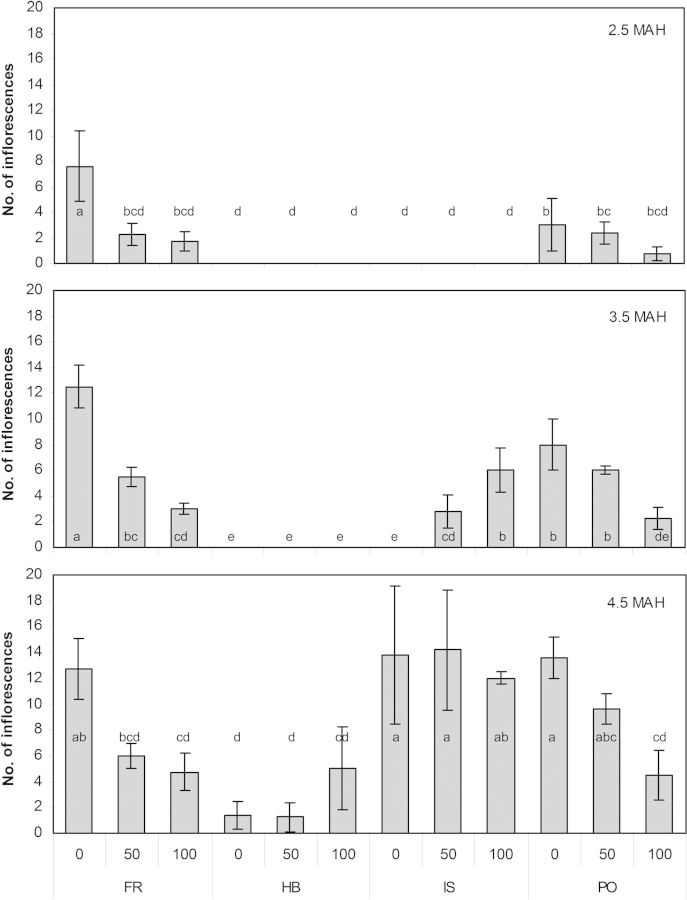
Effect of salinity on the flowering pattern of four *C. maritimum* genotypes. Number of inflorescences was counted at three time points after the onset of flowering (2.5, 3.5 and 4.5 MAH). Values denoted by different letters are significantly different, *P* < 0.05.

The influence of salinity on the flowering pattern was investigated in the four genotypes. The number of inflorescences was counted at three time points. Both Atlantic genotypes, FR and PO, preceded the Mediterranean genotypes at flowering onset time (Fig. 3), determined 2.5 MAH. Salinity significantly reduced the number of inflorescences in FR at this time point. Similarly, a reduction in the number of flowering branches with increasing salinity was observed for FR and PO.

One month later (3.5 MAH), genotype IS initiated flowering branches first in the saline treatments, while HB had still not flowered. After an additional 2 months (4.5 MAH), the IS genotype exhibited the maximum and its Mediterranean counterpart the minimum number of inflorescences, both regardless of the salt treatment.

### Evaluation of leaf constituents

Total soluble solids, which represent an estimate of the accumulation of compatible organic solutes and EC, a parameter indicative of mineral accumulation, were determined in leaf extracts (Table 1). Electrical conductivity values increased significantly with increasing salinity in all genotypes without major differences between them, pointing to general salt accumulation in the leaves. On the other hand, TSS significantly increased only in the Atlantic genotypes PO and FR; FR accumulated the highest TSS at the 100 mM salt treatment of all the genotypes.

**Table 1.** Effect of salinity on EC and TSS in four *C. maritimum* genotypes. Values denoted by different letters are significantly different, *P* < 0.05.

Leaf constituents	Salinity (mM NaCl)	FR	HB	IS	PO
EC (dS m^−1^)	0	19.8e	14.7f	14.8f	16.5f
	50	22.3d	28.0a	25.3bc	23.3d
	100	25.3bc	27.0ab	26.3ab	24.0cd
TSS (%)	0	4.5e	4.7de	5.2bcd	5.0cde
	50	5.5bc	5.3bc	5.0cde	5.7ab
	100	6.2a	5.0cde	5.2bcd	5.7ab

### Determination of ASC

**Figure 4.**
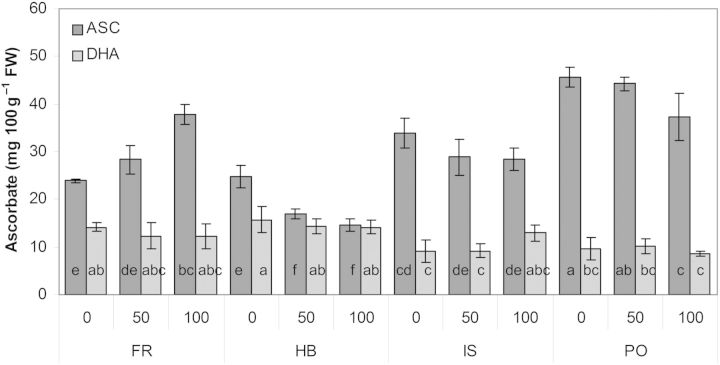
Effect of salinity level on ASC and DHA in four *C. maritimum* genotypes. Values denoted by different letters are significantly different, *P* < 0.05.

Total ASC concentration in the leaves was determined by two components, reduced ASC and DHA. Dehydroascorbate was not influenced by genotype or by salinity (Fig. 4). Ascorbate significantly increased in FR and decreased in HB, with the increase in salinity. Salinity had no effect on ASC in the genotypes IS and PO. The highest ASC concentration was found in PO at all salinity levels, reaching almost double that in HB.

### Total polyphenol determination

**Figure 5.**
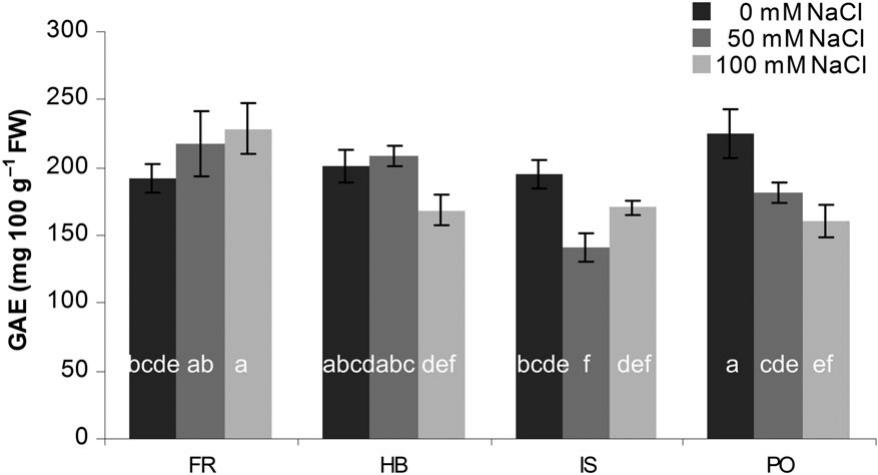
Effect of salinity level on total polyphenols, expressed as gallic acid equivalents (GAE), in leaves of four *C. maritimum* genotypes. Values denoted by different letters are significantly different, *P* < 0.05.

Leaf polyphenol concentration decreased with increasing salinity in all genotypes, except FR, where a significant increase was observed (Fig. 5). The most remarkable reduction was evident in genotype PO at 100 mM NaCl—30 % lower polyphenol concentration than the non-saline control. In the control treatment (no added salinity), there were no differences in polyphenol concentration between genotypes, but when grown under salinity polyphenol concentration in genotype FR exceeded that of all the others, particularly at the highest salt treatment.

### Determination of ureides

**Figure 6.**
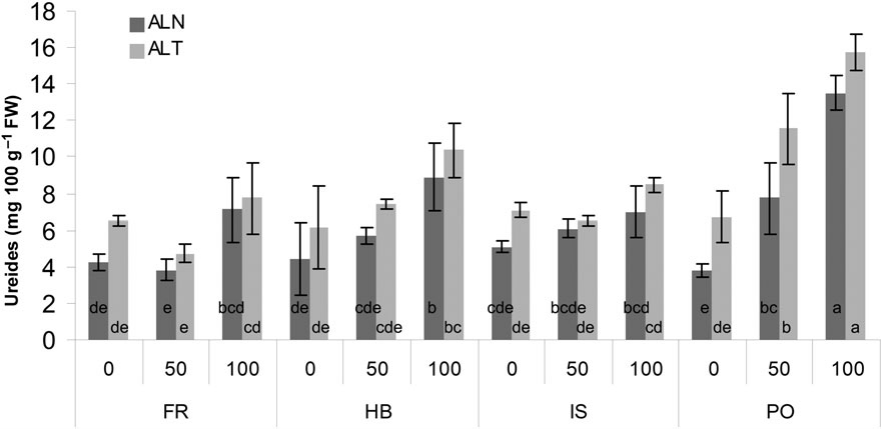
Effect of salinity on allantoin (ALN) and allantoate (ALT) levels in leaves of four *C. maritimum* genotypes. Values denoted by different letters are significantly different, *P* < 0.05.

During purine degradation, uric acid is catabolized into ureides ALN and ALT. In *C. maritimum* leaves, the ratio between ALN and ALT is ∼1 : 1 (Fig. 6). When plants were grown without salt there were no differences between the genotypes, but with salt application genotype PO differed significantly from its counterparts for both ureides, ALN and ALT (Fig. 6). Saline irrigation of 100 mM NaCl induced a 3.5-fold enhancement of ALN and a 2.3-fold enhancement of ALT, in genotype PO. In genotype HB, there was a significant increase of ALT, the more degraded ureide, at 100 mM NaCl, while in FR and IS leaf ureide concentration was not influenced by salinity.

### Fatty acid profile

A leaf fatty acid profile for the four *Crithmum* genotypes at three salinity levels was composed (Table 2). Total fatty acid concentration increased significantly with increasing salinity in HB and IS, but was unaffected in genotypes FR and PO. Most fatty acids were not influenced by salinity. Differences between genotypes, not related to salinity, were found in the content of omega-3 fatty acid 16 : 3ω3, which was significantly lower in genotype FR, while linoleic acid (18 : 2) was highest in FR when compared with all other genotypes.

**Table 2.** Effect of salinity on FAME profile in leaves of four *C. maritimum* genotypes. Values in the table are mean fatty acid (as % of total fatty acids) (*n* = 3). Values denoted by different letters are significantly different, *P* < 0.05.

FAME	Genotype											
	FR			HB			IS			PO		
	Salinity (mM NaCl)											
	0	50	100	0	50	100	0	50	100	0	50	100
16 : 0	19.9bc	19.2bc	18.4c	22.3a	20.2bc	21.1ab	19.0c	18.8c	19.0c	19.3bc	19.0c	18.9c
16 : 1	3.2bc	2.9bc	3.0bc	2.5c	3.0bc	2.6bc	4.1a	3.4ab	3.2bc	2.5c	2.6bc	2.9bc
16 : 1 pg	2.5e	2.4e	2.2e	3.3a	3.1ab	3.4a	2.7cd	2.8cd	2.9c	2.9bc	2.8cd	2.8c
16 : 2	0.5b	0.5Ab	0.6b	0.0e	0.3cd	0.5b	0.1de	0.5bc	0.7b	0.5b	0.6b	0.9a
16 : 3ω3	6.4b	6.4b	6.2b	8.6a	9.3a	9.9a	9.1a	9.5a	10.0a	9.8a	9.5a	9.5a
18 : 0	2.5ab	2.5ab	2.6a	2.4abc	2.0d	2.1cd	2.4abc	2.2bcd	2.3bcd	2.1cd	2.2bcd	2.4abc
18 : 1ω9	1.7bcd	1.6bcde	1.8bc	1.2cde	1.1e	1.2de	1.5bcde	1.8bc	2.0ab	2.0ab	1.8bc	2.4a
18 : 2	32.3a	33.2ab	33.9a	29.9cd	28.1ef	26.9f	29.1cde	29.4cde	28.8de	29.3cde	30.4c	30.0cd
18 : 3ω3	30.7a	31.3a	31.1a	29.2a	32.2a	31.4a	31.Aa	31.2a	30.7a	31.0a	30.2a	29.3a
Others	0.4cdef	0.0f	0.2def	0.6abcd	0.6abcd	1.0a	0.1ef	0.5bcde	0.6abcde	0.6abcd	0.9ab	0.7abc
Total FAME (%DW)	2.3bcd	2.2bcd	2.3bcd	1.9d	2.4abc	2.4abc	2.1cd	2.5ab	2.7a	2.7a	2.5ab	2.5abc
Total ω3	37.1c	37.7bc	37.3c	37.9bc	41.5a	41.2a	40.9ab	40.6ab	40.7ab	40.8ab	39.7abc	38.8abc

## Discussion

The comparison between four *C. maritimum* genotypes revealed differences in leaf appearance, plant habit and flowering pattern (Figs 1–3). *Crithmum maritimum* leaves were described by Atia *et al.* (2011) as fleshy and succulent, which was confirmed for all genotypes. The appearance of a broad or narrow leaf lamina was determined by the origin of the genotype and influenced its overall plant morphology. When used as ground cover for landscaping, plants with a compact growth habit and broad leaf appearance (in our case FR, HB, IS) may be of advantage, particularly because their biomass production was not negatively affected by the salt treatment (Fig. 2). Interestingly, in the Atlantic genotypes, FR and PO, the onset of flowering was early in the season regardless of salinity, but the number of inflorescences significantly decreased with the increase of salt concentration, indicating that those genotypes are salt sensitive during their reproductive phase. On the contrary, the late-season Mediterranean genotype IS showed a salt-induced onset of flowering (Fig. 3, middle) and no reduction in the number of inflorescences, thus demonstrating the long-term salt tolerance of this genotype, a characteristic that would be advantageous as a flowering ground cover suitable to ‘greenify’ beach-site areas where mainly saline irrigation is available (Lieth 2000). Growing halophytic species with attractive flowers, several leaf forms or growth patterns (e.g. *Aster tripolium*, *Atriplex* species, *Batis maritima*) also increases the interest in halophyte gardening and the use of new resources by florists (Lieth 2000). Mixing of different leaf types and seasonal variation in flowering may further increase the interest in combining *Crithmum* genotypes for saline landscaping.

*Crithmum maritimum* is a ‘salt-including’ halophyte. These types of halophytes compartmentalize Na^+^ and Cl^−^ in vacuoles after these ions are inevitably taken up by the roots (Ben Amor *et al.* 2005). All genotypes accumulated the salt in a similar manner, as indicated by increasing EC values (Table 1), but an adjustment of the osmotic balance by increasing sugars as an osmo-protective mechanism (Türkan and Demiral 2009), indicated here by TSS values, was observed only in the Atlantic genotypes FR and PO.

Plants exposed to abiotic stresses prevent oxidative damage by synthesizing antioxidant compounds, such as ASC, carotenoids, glutathione and tocopherol as well as antioxidant enzymes (Ashraf and Harris 2004; Ben Hamed *et al.* 2007). Ksouri *et al.* (2007) reported that salt-tolerant accessions of *Cakile maritima* were found to possess an enhanced antioxidant production capacity, associated with higher ASC and polyphenol content than in less tolerant accessions. Similarly, we found an increase in ASC in genotype FR, the most salt tolerant in respect to fresh and dry biomass production, with increasing salinity (Figs 2 and 4). *Crithmum maritimum* has previously been evaluated as a rich source of phenolic compounds, which was influenced by location, season and flowering stage (Maleš *et al.* 2003; Meot-Duros and Magné 2009). Similar absolute values of phenolics to those reported by Meot-Duros and Magné (2009) for leaves sampled in spring were determined for genotype FR, while genotype PO, representing the smallest phenotype with low biomass production, exhibited a significant lower total polyphenol and ASC concentration (Figs 2, 4 and 5).

The ureides ALN and ALT are well known to be important N storage and transport compounds in legumes (Smith and Atkins 2002; Zrenner *et al.* 2006). However, the metabolic role of ureides in non-legumes is not well known (Mazzafera *et al.* 2008). Nevertheless, it was recently demonstrated that ureides accumulate in non-leguminous plants exposed to stress such as salinity, high NH_4_^+^ concentrations, dark-induced senescence and normal senescence (Sagi *et al.* 1998; Brychkova *et al.* 2008; Ventura *et al.* 2010). Surprisingly, our results demonstrate an enhancement of ureide concentrations with increasing salinity only in genotypes PO and HB. Nevertheless, the leaf ureide concentration in the halophyte *C. maritimum* under control conditions (without salinity) was more than 20-fold higher than that found in *Salicornia* spp., highlighting the possible use of ureide for efficient N-storage, under elevated salt conditions (Ventura *et al.* 2011*a*, *b*).

Omega-3 fatty acids, which are a major constituent of plant lipids located in the chloroplast membrane, are known for their beneficial effects on human health (Simopoulos 2004). The presence of these essential fatty acids differed greatly among six tested plants with edible leaves, including four halophytes and two non-halophytic species; *C. maritimum* was found to have the highest concentration in leaves after *Portulaca oleracea* (Guil-Guerrero and Rodríguez-García 1999). Ben Hamed *et al.* (2005) demonstrated an increase in total lipids as response to irrigation with up to 100 mM NaCl in a Tunisian *C. maritimum* variant and pointed out the important role of sulfolipids in the salt tolerance of *C. maritimum,* probably through stabilization of photosynthetic processes.

The current comparison revealed differences in fatty acid concentration between the four genotypes within the tested salt range: FR and PO were not influenced by salinity, while HB and IS exhibited an increase in fatty acid concentration with increasing salinity (Table 2). Although the fatty acid concentration did not change with salinity in genotype FR, this genotype had the highest 18 : 2 and lowest 16 : 3ω3 values. The characteristic of restructuring membranes with less polyunsaturated acids during salt stress is suggested to protect against the oxidative effects of salt ions (Qureshi *et al.* 2013). The absolute fatty acid concentrations of all tested genotypes exceeded most of those reported by Ben Hamed *et al.* (2005), but were below those found by Guil-Guerrero and Rodríguez-García (1999) for plant materials collected in Spain, highlighting the importance of comparative studies of plant material from different origins.

The four genotypes differed in most of the parameters we investigated, including onset of flowering and leaf metabolites. Environmental adaptations at the natural saline habitats were probably the main reason for the differences; particularly, the characteristic of flower appearance could be classified according to the geographic origin (Mediterranean and Atlantic) of the plants. We can speculate that the early onset of flowering in the Atlantic plant group (FR and PO) was due to an effect of latitude, resembling the early flowering observed in *Salicornia* spp. from northern geographical regions (Ventura *et al.* 2011*b*). The differences in leaf appearance and growth habits of the genotypes (Figs 1 and 2) may indicate adaptation to the environmental conditions (climate, sea water salinity, soil type, distance to sea, tides) existing in their natural habitats that may be followed by the synthesis of their particular leaf metabolites. The combination of both plant morphology and leaf metabolites likely endowed the genotypes with their overall salt tolerance.

## Conclusions

Four *C. maritimum* genotypes from different origins (Mediterranean and Atlantic) were compared for their dual potential as ornamental plant and leafy vegetable under saline irrigation. Flowering onset seems to be dependent on parameters (e.g. origin) other than salinity, while a higher salt tolerance, determined by biomass enhancement, of the Atlantic genotype FR was correlated with increasing TSS, polyphenols and ASC. The exact role of ureides and fatty acids in *C. maritimum* genotypes in response to salinity still needs further investigation.

The parameters we evaluated are important criteria for genotype selection and development towards a multi-purpose halophytic crop for ornamental landscaping and as a leafy vegetable with nutritional potential.

## Sources of Funding

The research was supported by a grant from the Chief Scientist, Ministry of Agriculture and Rural Development, Israel, grant no. 857-0548-08.

## Contributions by the Authors

The research was conducted in M.S. laboratory. Y.V. and M.S. contributed to the main idea, data analysis and writing of the manuscript, M.M. carried out the experiments; Z.A. and R.O. supervised M.M. and I.K.-G. was responsible for the FAME evaluation.

## Conflicts of Interest Statement

None declared.
